# Isolation and proteomic profiling of urinary exosomes from patients with colorectal cancer

**DOI:** 10.1186/s12953-023-00203-y

**Published:** 2023-02-09

**Authors:** Ling Ma, Haijiao Yu, Yubing Zhu, Kaiyu Xu, Aimin Zhao, Lei Ding, Hong Gao, Man Zhang

**Affiliations:** 1 Department of Gastrointestinal Tumor Surgery, Beijing Shijitan Hospital Affiliated to Capital Medical University, Beijing, 100038 People’s Republic of China; 2 Department of Clinical Laboratory, Beijing Shijitan Hospital Affiliated to Capital Medical University, No. 10 Tieyi Road, Haidian District, Beijing, 100038 People’s Republic of China

**Keywords:** Colorectal cancer, Urine, Exosome, Mass spectrometry, Bioinformatics analysis

## Abstract

Exosomes in the body fluid are effective cell-derived membranous structures transferring various molecules to mediate intercellular communication. The expression of protein in the urinary exosomes from the colorectal cancer (CRC) patients could reflect the characteristics of tumorigenesis. The urinary exosomes with globular membrane structure, the size of 30 ~ 100 nm and positive expression of CD9, CD63 and CD81 were successfully isolated from 9 CRC patients and 3 heathy adults using the density gradient ultracentrifugation. Proteome profiles revealed by label-free liquid chromatography-tandem mass spectrometry (LC–MS/MS) indicated that several proteins were differentially expressed among different stages of CRC. Compared with normal controls, 67 proteins in CRC urinary exosomes were upregulated and 74 proteins were downregulated. The bioinformatics analysis revealed the decreased proteins were related to ESCRT III complex disassembly. The CHMP family was further determined to be the hub of interaction network of proteins enriched in ESCRT signaling. The significant decrease of CHMP4A, CHMP4B, CHMP2A, CHMP2B and CHMP1B were respectively found in the total CRC group and distant metastasis group compared with NC group. Moreover, the CEACAM family also showed significant aberrant changes in the urinary exosomes of CRC patients. The CEACAM7 and CEACAM1 were increased in the CRC patients compared with healthy individuals (*P* < 0.05). Significant changes of proteomic profile could be found in the urinary exosomes in the CRC patients. The differential expressed urinary exosomes derived proteins showed potential usage in diagnosis and prognosis of CRC.

## Background

Although the long-term decline in mortality has been slowed for colorectal cancer (CRC), it still ranks as the third most common cancer and the third leading cause of cancer related death in both men and women according to the latest data from American Cancer Society. The prognosis of CRC patients is closely related to the tumor stage. The five-year relative survival of different stages of CRC were apparently varied, with 90% for localized disease, 72% for regional disease, and only 14% for distant metastatic CRC [1]. To explore efficient and reliable measurements and biomarkers for cancer screening, earlier diagnosis, appropriate clinical surveillance and prognostic evaluation are meaningful to reduce cancer-specific mortality rate. Several traditional serum tumor markers are widely used as the indicators for screening, diagnosis and post-operative surveillance of gastrointestinal cancer, but with insufficient sensitivity and specificity.

Extracellular vesicles (EVs) are membranous particles which were released by cells to the extracellular environment to executive communication function through carrying diverse types of cargos including DNA, RNA, lipids and proteins. It is reported that EVs have been isolated from diverse biological fluids, such as blood, urine, saliva, breast milk, amniotic fluid, ascites, cerebrospinal fluid, bile, and semen [2]. EVs are usually composed of both exosomes and shed microvesicles (sMV). The secretion machineries of exosomes and sMV are different. Moreover, the diameter of isolated exosomes observed should be in the size ranging from 30 to 100 nm, and the size of sMV is larger than exosomes, with the diameter ranging from 100 nm to 1 μm [3]. In this study, we will focus on the EVs with the diameter less than 100 nm and present typical markers of exosomes.

As important mediators of intercellular communication, exosomes released by various types of cells participate in the regulation of physiological process and tumorigenesis. The tumor-derived exosomes were reported to be instrumental in promoting local development and metastatic dissemination through increasing local motility of tumor cells [4] or directly seeding tumor-draining lymph nodes before further migration of tumor cells themselves [5]. They could also induce premetastatic niche formation via immunosuppression, angiogenesis, stromal cell remodeling, and oncogenic reprogramming [6]. Moreover, the lipid bilayer membrane of exosomes could protect their cargo from RNases and proteases [7]. It is meaningful to explore the tumor-specific contents in the circulating exosomes as the biological markers and therapeutic targets. The serum exosomes have extensively investigated in various types of diseases. Significant expressed proteins, microRNAs and noncoding RNAs had revealed potential diagnostic and prognostic value of different cancers.

In 2004, Pisitkun et al. successfully isolated exosomes in the urine sample for the first time. They identified 295 proteins including multiple protein products of genes which were reported to be responsible for renal and systemic diseases [8]. Since then, urinary exosomes were widely studied in the diseases of urinary system. There was massive research blank about the correlation between the urinary exosomes and digestive system diseases, such as CRC. A considerable quantity of circulating exosomes would eventually excrete in urine, including the physiological changed exosomes induced by the disease situation. Moreover, urine was the body fluid with maximum amount which can be easily obtained in a non-invasive way. It is feasible and operational to isolate exosomes in the urine sample of CRC patients and analyze the contents of exosomes to find out whether there were differential expressed proteins or RNAs associated with CRC.

In the present study, we tried to isolate urinary exosomes from CRC patients with different stages and healthy individuals. Then the proteome profiles of exosomes from different groups were analyzed and compared to explore the significant differentially expressed proteins and signaling pathways.

## Materials and methods

### Patients and urine samples

Nine patients with CRC diagnostic by pathology of histology in Beijing Shijitan Hospital from January 2020 to January 2021. These patients had no history of other malignancies, and had not received adjuvant chemoradiotherapy before surgery. Based on the clinicopathological indexes, they were divided into three groups, such as local cancer without metastasis (C), locally advanced cancer with lymph nodes metastasis (CLN) and cancer with distant metastasis (CM), three subjects for each group. Three healthy people were included as controls (NC). Written informed consents were obtained from all subjects. The study was approved by the Ethics Review Committee of Beijing Shijitan Hospital.

Fifty Milliliter morning urine from all the participants were collected in sterile containers for exosome extraction. It should be stored at -20 ℃ before following processing.

### Isolation of EVs from urine

The EVs in urine were isolated by the density gradient ultracentrifugation. After rapid melting under room temperature, the urine sample was centrifuged at 2000 × g, 4 ℃ for 30 min to remove the floating cells and other impurities, then at 10,000 × g, 4 ℃ for 30 min to remove the macrovesicles. Supernatant was filtered (0.45 mm, Merck Millipore) to remove contaminating microvesicles and cell debris, and then centrifuged at 100,000 × g, 4℃ for 70 min twice. The precipitate was resuspended in the 100 μl pre-cooled Phosphate Buffered Saline (PBS) and stored at -80℃.

### Transmission electron microscopy

The EVs were observed using the transmission electron microscopy (TEM) (Hitach, Japan). 10 μl suspension was plated on the TEM grid and precipitated for 1 min. Then the supernatant was removed by filter paper. 10 μl uranyl acetate was transferred to the grid and precipitated for 1 min. After the supernatant was removed by filter paper, the grid was dried under room temperature for few minutes and observed with HT-7700 transmission electron microscope at 100 kV.

### Quantitative analysis of EVs

The size and concentration of microparticles were determined by Flow NanoAnalyzer (NanoFCM, Xiamen, China). 10 μl suspension was diluted into 30 μl and then measured by NanoFCM according to the manufacturer’s instruction. All the data were analyzed with relative software. Three independent experiments were performed.

### Nanoparticle flow cytometry

The tetraspanins at the surface of exosomes, CD9, CD63 and CD81 were commonly used as biomarkers. NanoFCM could also provide high sensitivity flow cytometry for nanoparticle analysis. The 488-nm laser diode was selected as the laser to illuminate the sample. The detection channels were side-scattered light and Fluorescein Isothiocyanate (FITC) fluorescence channel respectively. The EVs were incubated with FITC conjugated anti-CD9, CD63, CD81 and IgG. Then the diluted sample that yielded 100–150 detection events per second was analyzed by NanoFCM.

### Exosomal protein extraction and filter-aided sample preparation

The remaining 80 μl supernatant mixed with 400 μl lysis solution (7 M urea, 2 M thiourea, 0.1% protease inhibitor and 65 mM DTT) was processed with ultrasonication in ice bath (70 W, 5 s, 3–5 times) and placed on ice for 40 min. After centrifugation at 5000 × g, 4 ℃ for 30 min, the supernatant was collected for filter-aided sample preparation (FASP). The protein sample was diluted with 200 μl UA (8 M urea in 0.1 M Tris, PH 8.5) and then reduced with 20 mM DTT for 4 h at 37 °C. The protein was alkylated with 50 mM iodoacetamide (IAA) for 30 min in the dark, and the alkylation reaction was quenched with 200 μl 5 Mm DTT. After centrifugation at 14,000 × g, 15 min for twice, 200 μl ABC solution (50 mM NH_4_HCO_3_ in water) was added to the sample and the centrifugation process was repeated. 100 μl ABC solution containing trypsin (trypsin: protein = 1: 50) was added to the membrane and digested overnight at 37 ℃. The next day, peptides were eluted from the membrane by centrifuging for 15 min at 14,000 × g. 100 μl of water was used for a second elution. Samples were dried using a vacuum centrifuge.

### Label-free liquid chromatography-tandem mass spectrometry (LC–MS/MS)

The LC–MS/MS was performed using the Easy-nLC 1200/Orbitrap Fusion Lumos Mass Spectrometer (ThermoFisher Scientific, Bremen, Germany). Peptides were dissolved in 0.1% formic acid (FA) and separated with the autosampler. Peptide mixture were loaded on a trap column (PepMap C18, 100 Å, 100 μm × 2 cm, 5 μm), and then separated on the analytical column (PepMap C18, 100 Å, 75 μm × 50 cm, 2 μm) in mobile phase A [ACN-H2O-FA (1.9; 98; 0.1, v/v/v)] and mobile phase B [ACN-H2O-FA (98;1.9; 0.1, v/v/v)]. The MS instrument was operated in a data dependent acquisition mode (DDA). The conditions of gradient elution were as follows: 6–9% buffer B for 0–8 min, 9–14% buffer B for 8–24 min, 14–30% buffer B for 24–60 min, 30–40% buffer B for 60–75 min, 40–95% buffer B for 75–78 min, 95% buffer B for 78–85 min, and then changed to 6% buffer B within 1 min and equilibrated for 4 min. Spectra were scanned over the m/z range 400–1500 Da at 35,000 resolution. 30 s exclusion time and 30% normalization collision energy were set at the dynamic exclusion window.

### Data analysis

RAW files were extracted was performed using the Maxquant software (version 1.5.2.8, Germany). Label-free quantitation analysis (LFQ) was performed according to the intensity based absolute quantification (iBAQ). The parameters were set as follows: fully tryptic peptides with ≤ 2 missed cleavages were permitted; carbamidomethylation (C) was as fixed modifications, oxidization (M) and acetyl (protein N-term) were as variable modifications; the charge state of the peptides were set from 2 to 7. The cut-off of global false discovery rate (FDR) for peptide and protein identification was set to 0.01.

Functional annotation analysis of differentially expressed proteins was determined by gene ontology (GO) enrichment using the DAVID online tool (http://david.abcc.ncifcrf.gov/) and Kyoto Encyclopedia of Genes and Genomes (KEGG) using the WEB-based Gene SeT AnaLysis Toolkit (http://bioinfo.vanderbilt.edu/webgestalt/). The Search Tool for Recurring Instances of Neighbouring Genes (STRING) database (http://string-db.org/) was used for network analysis.

### Statistical analysis

All data were expressed as mean ± standard deviation (s.d.). The results were statistically analyzed using GraphPad Prism 5 statistical software (San Diego, CA). Differences between groups were determined using Student’s t-test. *P*-value of < 0.05 was considered statistically significant.

## Results

### Characterization of urinary EVs from CRC patients and healthy individuals

**Fig. 1:**
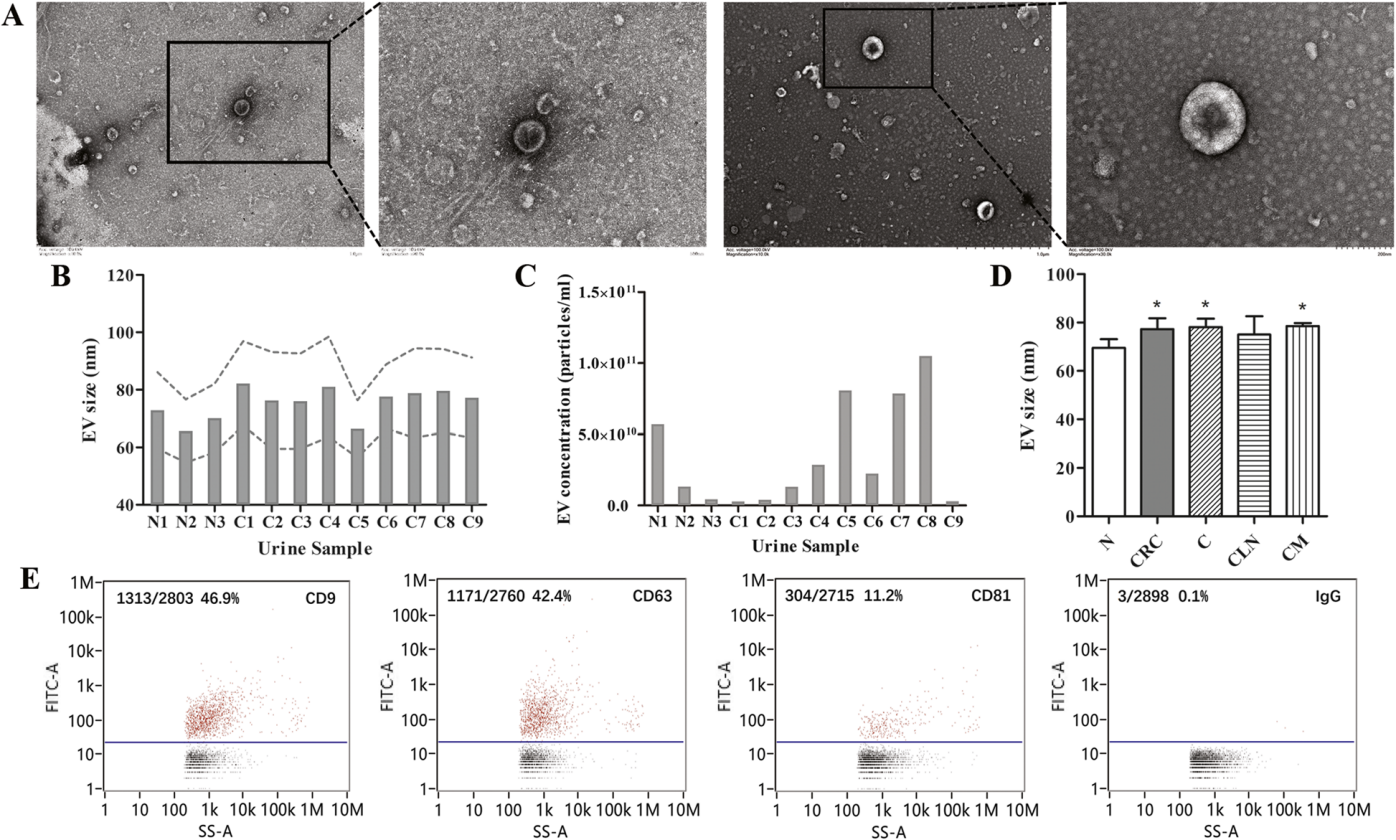
The characterization of EVs isolated from urine. **A** Representative TEM images of EVs isolated from the urine samples. The size (**B**) and concentration (**C**) of EVs measured by NanoFCM of each urine sample. **D** Comparison of the size of EVs isolated from the CRC, C, CLN, CM and NC group. **D** Dot-plots of EVs staining with FITC-conjugated antibody specific to CD9, CD63 or CD81 compared with IgG. Results were expressed as mean ± s.d., *P < 0.05

EVs were successfully isolated from the urine samples of 9 CRC patients and 3 healthy individuals via the density gradient ultracentrifugation. TEM showed that these microvesicles were globular particles with size of 30 ~ 100 nm (Fig. 1A). The diameter of EVs were between 60 ~ 80 nm (Fig. 1B), and the concentration were between 2 × 10^9^ ~ 1 × 10^11^ particles/mL (Fig. 1C). The size of EVs isolated from CRC group (77.25 ± 4.56 nm), C group (78.16 ± 3.47 nm) and CM group (78.58 ± 1.21 nm) were significantly increased compared with N group (69.53 ± 3.62 nm) (*P* < 0.05) (Fig. 1D). The positive expression of CD9, CD63 and CD81 were determined by nanoparticle flow cytometry (Fig. 1E). Given that, the EVs extracted from the human urine sample reached the specifications for exosomes. Density gradient ultracentrifugation was proven to be an effective method to isolate homogeneous exosomes from human urine samples.

### Proteomic profiling and comparison of urinary exosomes from different CRC group

**Fig. 2:**
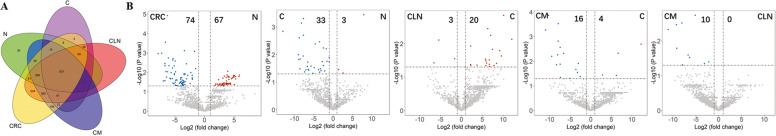
Proteomic profiling and comparison of urinary exosomes from different CRC groups determined by LC–MS/MS. **A** Venn diagram of urinary exosome-derived proteins detected in different CRC groups (CRC, C, CLN and CM) and healthy people (N). **B** Volcano plot of urinary exosome-derived proteins detected and compared in different CRC groups (CRC, C, CLN and CM) and healthy people (N). Proteins in upper left and right quadrants are significantly differentially expressed

The proteomic analysis of the urinary exosomes based on LC–MS/MS explored 2277 proteins from the twelve samples. Specifically, a total of 1359 proteins, 2246 proteins, 1029 proteins, 1986 proteins and 1813 proteins were identified in N, CRC, C, CLN, CM group respectively (Fig. 2A). Several significantly differentially expressed proteins were recognized between groups showed by volcano plot (Fig. 2B).

**Fig. 3:**
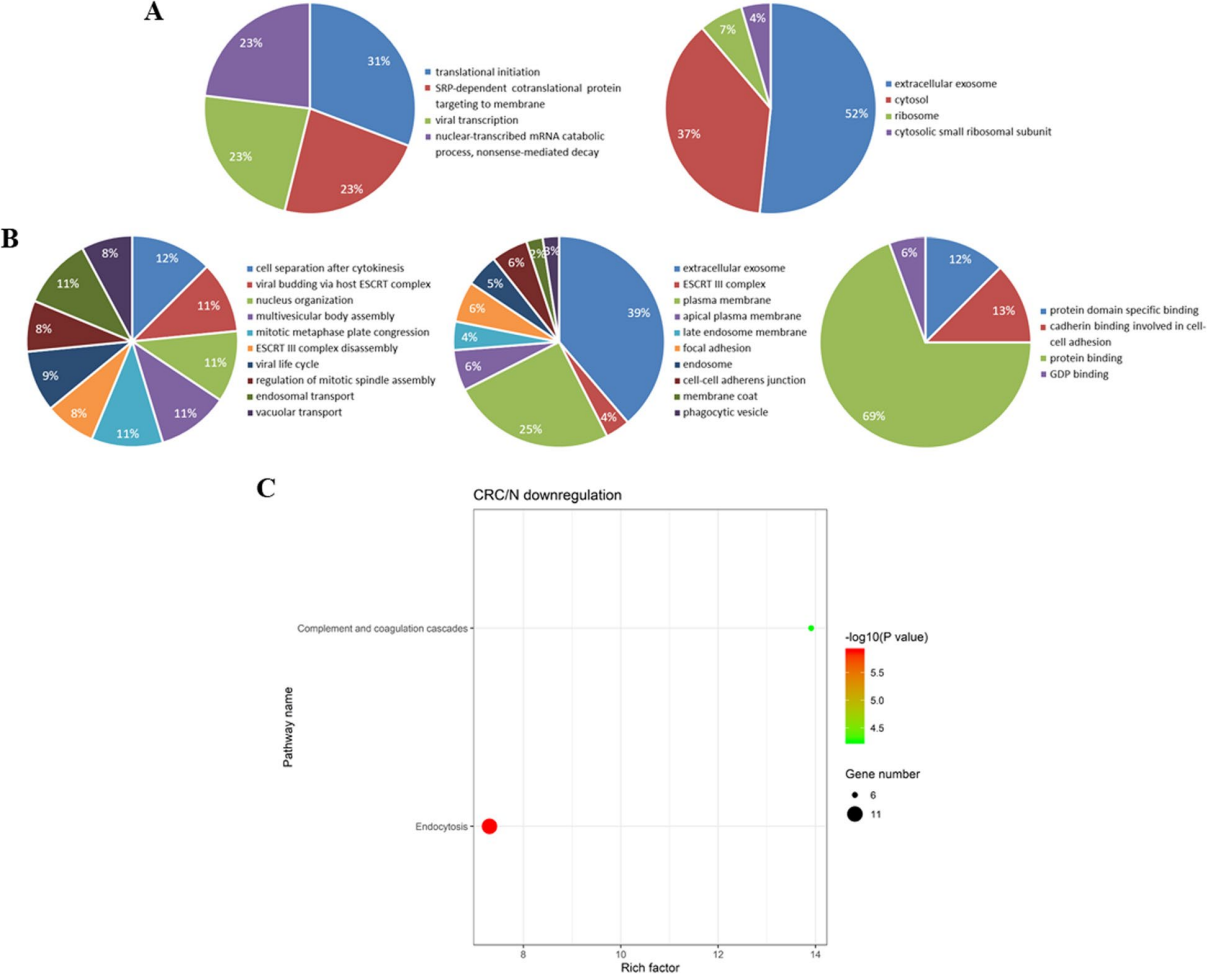
Bioinformatics analysis of differentially expressed proteins identified from the urinary exosomes between CRC and NC group. GO analysis of the significantly upregulated proteins (**A**) and downregulated proteins (**B**) in the aspects of biological process, cellular components and molecular function, respectively. (**C**) KEGG bubble diagram of the significant enriched pathways targeted by the downregulated proteins in the urinary exosomes from CRC group compared with NC group

Further bioinformatics analysis of GO and KEGG analysis were performed on the 67 upregulated proteins (fold change > 2) and 74 downregulated proteins (fold change < 0.5) in the CRC group compared with NC. It was noting that the downregulated proteins in CRC versus N were mainly related to the cell component of ESCRT III (endosomal sorting complex required for transport III) complex and biological process of ESCRT III complex disassembly revealed by GO analysis (Fig. 3A), and the most enriched signaling pathway was endocytosis showed by KEGG analysis (Fig. 3B). ESCRT III complex was involved in degradation of surface receptor proteins and formation of endocytic multivesicular bodies (MVBs). It was believed that the component interaction of ESCRT complexes was essential for endocytosis-dependent activity. Loss of any ESCRT component resulted in abnormal function in endocytosis [9].

### Identification of significant changed urinary exosomal proteins in CRC patients compared with healthy individuals

**Fig. 4:**
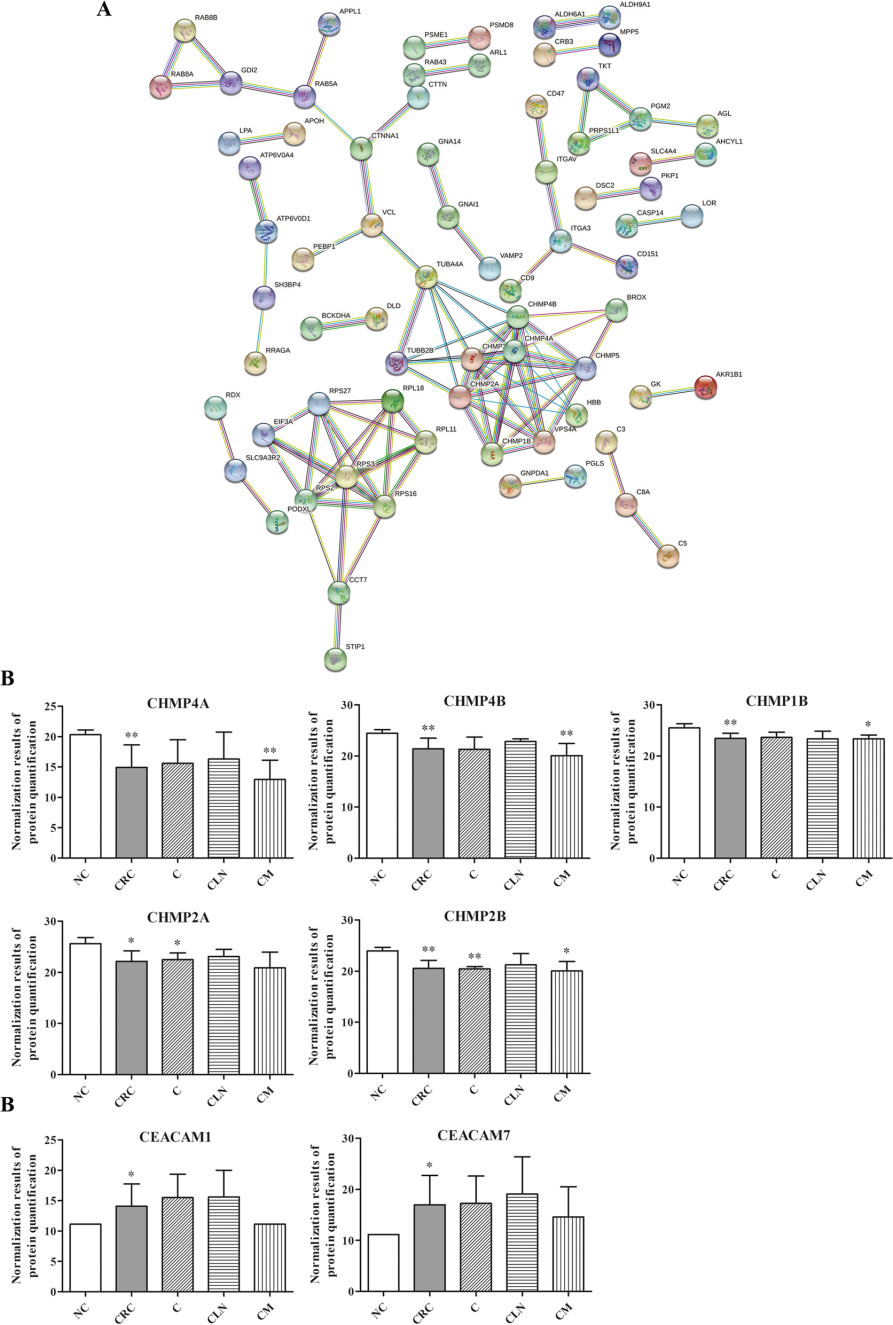
Identification of significant changed proteins in the urinary exosomes from CRC patients compared with healthy individuals. **A** The protein interaction network was generated using the STRING database. Comparison of the protein level of CHMPs (**B**) and CEACAMs (**C**) in different CRC groups compared with NC group. Quantification of representative differentially expressed proteins in different groups. Results were expressed as mean ± s.d., *n* = 3, *P < 0.05, **P < 0.01

The further analyzation via STRING database revealed that the chromatin-modifying protein/charged multivesicular body protein (CHMP) family was the hub of interaction network of proteins enriched in ESCRT signaling, especially the CHMP4A, CHMP4B, CHMP2A, CHMP2B and CHMP1B (Fig. 4A). The quantitative analysis of target proteins was further evaluated. The level of above CHMP members were significantly decreased in CRC patients than healthy individuals. Moreover, the significant decrease of CHMP4A, CHMP4B and CHMP2B were also found in the distant metastasis group (*P* < 0.05) (Fig. 4B).

Moreover, we discovered that the members of carcinoembryonic antigen (CEA) family represented remarkable changes in the differential expressed proteins in the urinary exosomes from CRC patients. The protein level of CEACAM7 and CEACAM1 increased 56.27-fold and 7.71-fold in CRC urinary exosome compared with NC group (*P* < 0.05) (Fig. 4C).

## Discussion

To explore the specific, efficient and convenient measurements and biomarkers for CRC screening, earlier diagnosis, appropriate clinical surveillance and prognostic evaluation was meaningful to reduce cancer-specific mortality rate. Besides the imaging examination, the traditional serum molecules, such as CEA, cancer antigen 19–9 (CA19-9) and cancer antigen 72–4 (CA72-4) were applied as tumor biomarkers but with insufficient sensitivity and specificity. As the technology of extracting exosomes from body fluids matured, to identify the significantly differentially expressed molecules had been the focus of recent researches. Released by various types of cells including tumor cells, the exosomes carried diverse cargos into the circulation undertaken the task to mediate communications, deliver signals and discharge wastes. It was believed that exosomes participated in growth and metastasis of tumors by regulating the immune response, blocking the epithelial-mesenchymal transition, promoting angiogenesis and developing chemotherapy resistance. Exosomes in liquid biopsies could be used as non-invasive biomarkers for early detection and diagnosis of cancers. Because of their amphipathic structure, exosomes were natural drug delivery vehicles for cancer therapy [10].

Previously, proteomics analysis had been performed on the CRC tissues and cell lines [11]. Chen et al. firstly explored differential expressed protein content in the serum purified exosomes from CRC patients compared with healthy volunteers and revealed that these proteins were involved in processes that modulated the pretumorigenic microenvironment for metastasis. However, the blood volume for exosomes extraction and analysis was as least 12.5 ml [12]. The presence of highly expressed proteins, such as albumin in the blood could be obvious interference to identify the tumor-specific proteins with relatively low abundant [13]. As a part and the end of circulation, urine could be the non-invasive sample sauce providing large amounts of exosomes containing functional proteins of importance in the process of tumorigenesis. In our experiment, sufficient concentration of exosomes could be obtained from 50 ml urine sample. Although urinary exosomes had a direct relation with diseases of urinary system, they could reflect the status and characteristics of other diseases.

In the present study, the mass spectrometry of urinary exosomes from CRC patients revealed significant changed proteome profiles compared with healthy individuals. The bioinformatics analysis based on the significant decreased proteins were mainly correlated with related to ESCRT III complex disassembly and the pathway of endocytosis. The ESCRT machinery was responsible for receptor down-regulation and retroviral budding. With the help of ESCRT I, II and III complex, countless signaling receptors were either recycled back to the cell surface (receptor sequestration) or sorted into the MVB pathway for lysosomal degradation (receptor down-regulation). ESCRT I played a critical role in ESCRT-mediated receptor down-regulation in tumorigenesis, such as aberrant EGFR degradation. However, none of the ESCRT III subunits had so far been implicated in cancer [14]. ESCRT-III complex was required for the formation and abscission of intraluminal endosomal vesicles [15]. ESCRT-III proteins, also called CHMPs, consisted of 11 isoforms in humans. Some members of CHMP family significant decreased in the urinary exosomes from the CRC patients revealed by our research. These proteins were correlated with regulation of the abscission of cytokinesis and endocytic trafficking. For example, CHMP4A, 4B played important role in membrane deformation and vesicle/tube formation, and CHMP2A, 2B related to membrane constriction/fission. There were barely studies exploring the function of CHMPs in malignancies. CHMP1A had been reported as a tumor suppressor in renal cell cancer via proliferation inhibition [16]. It also mediated the growth inhibitory activity of all-trans retinoic acid in human pancreatic cancer cells via regulation of CRBP-1 [17]. The significant downregulation of CHMPs in the urinary exosomes of CRC patients might suggest the inadequate exosome formation in the tumorigenesis or loss of their original ability to inhibit cancer through certain signaling pathways. These proteins could be the targets provide a base for the further research.

The traditional tumor marker CEA was firstly isolated from the fetus and colorectal cancer tissues but not the healthy adult colorectum in 1965. The twenty-nine members of CEA family fell mainly into two categories: carcinoembryonic antigen-related cell adhesion molecule (CEACAM) and pregnancy specific-glycoprotein (PSG). Generally, the CEACAMs were anchored to the cellular membrane and PSG could be secreted to the blood. Recognized as biomarkers for many types of malignant tumors, the CEA family were verified to be involved with tumorigenesis, cell adhesion, tumor angiogenesis, molecular signal transduction and tumor immunity. Based on the previous study, the primary members CEACAM1 and CEACAM7 were recognized as tumor suppressors, while CEACAM5 and CEAMCAM6 were upregulated in almost 50% types of tumors and functioned as oncogenes. However, the role of above molecules in a certain kind of cancer had been unfixed and controversial. As an example, CEACAM1 were reported to be aberrant upregulated in colorectal adenoma and adenocarcinoma, and the protein intensity was correlated with the TNM staging [18]. Re-expression of CEACAM1 long cytoplasmic domain isoform could promote invasion and migration of CRC [19]. In accordance with that, we noticed that the CEACAM family also showed significant changes in the urinary exosomes of CRC patients exploring the mass spectrum analysis data. The CEACAM7 and CEACAM1 were significantly overexpressed in the urine samples of CRC patients compared with NC group (*P* < 0.05). These results were conflicted with part of previous studies. Besides the undiscovered oncogenic function of CEACAM1 and CEACAM7 in CRC, another hypothesis could be put forward that the aberrant expression of CEACAMs in urine might be the surplus which were eventually eliminated from the body. The consumption of CEACAMs actually reflected the true level of these molecules in the circulation induced by the malignancies. In the further research, the expression and correlation of CEACAMs in serum and urine should be examined and compared. The CEACAMs level in different body fluid could become the indicators of CRC tumor progression.

## Conclusion

We proposed urinary exosomes as a convenient and effective source for identifying non-invasive biomarkers of cancer and other diseases of different systems. The formation and loading cargos of exosomes were significantly affected by CRC tumorigenesis. The differentially expressed CHMPs and CEACAMs in the urinary exosomes could become potential tumor markers for CRC. Moreover, joint detection of CHMPs and CEACAMs in both serum and urine could provide meaningful information for diagnosis and prognosis of CRC.

## Data Availability

The data that support the findings of this study are available from the corresponding author upon reasonable request.
